# Lightweight LSTM-Based Adaptive CQI Feedback Scheme for IoT Devices

**DOI:** 10.3390/s23104929

**Published:** 2023-05-20

**Authors:** Noel Han, Il-Min Kim, Jaewoo So

**Affiliations:** 1 Department of Electronic Engineering, Sogang University, Seoul 04107, Republic of Korea; 2 Department of Electrical and Computer Engineering, Queen’s University, Kingston, ON K7L 3N6, Canada

**Keywords:** channel quality indicator feedback, long short-term memory, lightweight model, modulation and coding scheme, feedback overhead

## Abstract

As the number of Internet of things (IoT) devices increases exponentially, scheduling and managing the radio resources for IoT devices has become more important. To efficiently allocate radio resources, the base station (BS) needs the channel state information (CSI) of devices every time. Hence, each device needs to periodically (or aperiodically) report its channel quality indicator (CQI) to the BS. The BS determines the modulation and coding scheme (MCS) based on the CQI reported by the IoT device. However, the more a device reports its CQI, the more the feedback overhead increases. In this paper, we propose a long short-term memory (LSTM)-based CQI feedback scheme, where the IoT device aperiodically reports its CQI relying on an LSTM-based channel prediction. Additionally, because the memory capacity of IoT devices is generally small, the complexity of the machine learning model must be reduced. Hence, we propose a lightweight LSTM model to reduce the complexity. The simulation results show that the proposed lightweight LSTM-based CSI scheme dramatically reduces the feedback overhead compared with that of the existing periodic feedback scheme. Moreover, the proposed lightweight LSTM model significantly reduces the complexity without sacrificing performance.

## 1. Introduction

Internet of things (IoT) devices have been widely used in many fields, and they are estimated to increase in number to 5.5–12.6 trillion worldwide by 2030 [1]. As the number of IoT devices exponentially increases, the central station or base station (BS) should efficiently manage limited radio resources. The BS needs to know the channel state information (CSI) of the devices to efficiently manage the radio resources. Hence, the BS requests the devices to report their channel quality indicator (CQI), where the CQI is a 4-bit value that aims to reflect the channel state [2,3]. The BS determines the modulation and coding scheme (MCS) based on the CQI reported by the IoT device. The MCS strongly influences throughput because it combines the modulation and code rate according to the channel condition. Many studies have been conducted using adaptive modulation and coding (AMC) to obtain high throughput [4,5,6].

The CQI reporting incurs feedback overhead because the reporting data are transmitted over a low-speed feedback channel. However, if the device does not frequently report its CQI, the BS cannot accurately determine the MCS according to the channel condition, which results in performance degradation. Conversely, if the device frequently reports its CQI, the BS can accurately determine the MCS according to the channel condition, but the feedback overhead is large. Hence, the devices need to report their CQI aperiodically based on channel conditions to achieve a trade-off between the accuracy of the MCS selection and the feedback overhead.

In 5G systems, the two kinds of feedback mode are periodic and aperiodic feedback [3]. Some researchers have mathematically analyzed the feedback interval [7,8,9]. The authors of [7] mathematically analyzed the optimal feedback interval that maximizes the average received power. The authors of [8] mathematically analyzed the feedback interval that maximizes the bidirectional transmission throughput. The authors of [9] calculated the CSI-dependent interval and used adaptive modulation to obtain higher energy efficiency and throughput. However, the work of [7,8,9] focused on finding intervals of periodic rather than aperiodic feedback. Hence, there is a limit to reduce the number of feedback transmissions.

Machine learning (ML) has been applied to various fields and has led to substantial performance improvements. The 3GPP Release 18 standard applied artificial intelligence (AI)/ML for a new radio (NR) air interface [10]. Many researchers have focused on compressing the CSI feedback data by using a convolutional neural network (CNN)-based model, where the CNN-based CSI feedback consists of the CSI compression at the terminal and the reconstruction at the BS [11,12,13]. The authors of [11] introduced a CNN-based feedback model called “CsiNet”, in which an encoder and decoder model were introduced for CSI feedback. The CsiNet model evolved into CRNet and CsiNet+ with improved performance [12,13]. The CRNet outperforms the CsiNet without any extra flops by using convolution factorization and the cosine learning rate scheduler. The CsiNet+ improves the performance but it significantly increases the complexity due to the increase in the convolutional kernel size. Other researchers proposed autoencoder-based CSI feedback to improve the accuracy of the CSI feedback with feedback errors [14,15,16]. However, the work of [11,12,13,14,15,16] focused on reducing the amount of feedback data in the periodic feedback. Moreover, the conventional ML structures are too complicated to be used in IoT devices because IoT devices have lightweight processors and small memory capacities.

Some recent studies have been conducted to reduce the complexity of the neural network in CNN-based CSI feedback [17,18,19,20,21]. The ENet proposed in [17] reduced the complexity by exploiting the correlation of the real and imaginary part of CSI while improving the performance. The CLNet proposed in [18] reduced the complexity by integrating the real and imaginary parts with a spatial-wise attention block. The authors of [19] proposed two CSI feedback models: One is ConvCsiNet based on a CNN autoencoder, which improves the reconstruction performance, and the other is ShuffleCsiNet based on a lightweight ConvCsiNet, which saves memory space and computing power. The authors of [20] proposed an adaptive lightweight CNN-based CSI feedback, where the network adaptively finds the compression ratio of feedback data and reduces the complexity of the decoder by about 38.2% in comparison with the CsiNet. The CVLNet proposed in [21] reduced the complexity by using complex-valued convolutions in the CNN-based CSI feedback. However, the work of [17,18,19,20,21] also focused on compressing the feedback data, and moreover they failed to reduce the number of feedback transmissions because the terminal periodically reports its CSI.

Specifically, recurrent neural networks (RNNs) have been applied to CSI feedback in order to exploit the temporal correlation of wireless channel [22,23,24,25,26,27,28,29]. Existing work has demonstrated that RNNs can provide efficient CSI feedback and reconstruction for time-varying channels [22,23,24,25,26]. However, the number of parameters in RNN layers for CSI compression and reconstruction is generally too large. Although other work attempts to reduce RNN size [26,27], most competitive models still require very large parameters. In recent studies, MarkovNet, proposed in [28], and CoCsiNet, proposed in [29], improved CSI recovery accuracy with reduced model size. However, the work of [22,23,24,25,26,27,28,29] also focused on compressing the feedback data and neglected the feedback delay.

Some researchers predicted the channel state using the past channel state to solve the problem of the CSI quickly becoming outdated due to device mobility of feedback delay [2,30,31,32]. The authors of [2,30] deployed machine learning to resolve the CQI mismatch problem caused by outdated CSI. The authors of [31] proposed a deep reinforcement learning-based adaptive modulation (DRL-AM) scheme, in which the current CSI is predicted from outdated values. The authors of [32] integrated an LSTM model and a deep Q-network to overcome the outdated CSI problem for underwater acoustic communication with feedback delay. However, the work of [2,30,31,32] failed to reduce the number of feedback transmissions because the terminal periodically reports its CSI, although they took into account the feedback delay or outdated CSI. Moreover, the complexity of ML is too high to be implemented in the lightweight IoT device. Recent work of [33] proposed aperiodic CSI feedback based on the deep neural network (DNN)-based channel prediction, where the terminal decides whether or not to feed back its CSI relying on the DNN-based channel prediction. However, the work of [33] did not take into account the feedback delay, and moreover, applied a classical DNN rather than an LSTM model in time-series forecasting.

In this paper, we propose a lightweight LSTM-based CQI feedback scheme for IoT devices. Most previous studies have the following limitations. First, most previous studies have focused on compressing the amount of CSI feedback data in the ML-based CSI feedback. Moreover, the number of parameters in the ML model is generally too large. Second, most previous studies have failed to reduce the number of feedback transmissions because they assumed periodic feedback. Third, some studies considered the feedback delay, but most previous studies did not consider the feedback delay. In this paper, the above three problems are overcome. The contributions of this paper are as follows: First, we develop a lightweight LSTM model by decomposing the matrices of the conventional LSTM model using singular value decomposition (SVD) and applying dimensionality reduction. Hence, the proposed LSTM can significantly reduce the complexity. Second, we propose a lightweight LSTM-based CQI feedback scheme for IoT devices, where the IoT device aperiodically reports its CQI to the BS relying on LSTM-based channel prediction. Hence, the proposed LSTM-based aperiodic CQI feedback scheme can dramatically reduce the number of feedback transmissions in comparison with conventional periodic CQI feedback schemes. Third, we evaluate the performance of the proposed LSTM-based CQI feedback scheme under the feedback delay channel. The simulation results show that the proposed lightweight LSTM model shows equivalent performance to the conventional LSTM model despite reducing the complexity by half. The rest of this paper is organized as follows: Section 2 presents the system model and the AMC scheme. Section 3 introduces the proposed lightweight LSTM model and the LSTM-based CQI feedback scheme. Section 4 provides the simulation results and Section 5 concludes this paper.

## 2. System Model

### 2.1. Channel Model

We consider a single cell network, where IoT devices estimate the channel state by using the reference signal periodically broadcast by the BS. The BS and IoT devices have a single antenna. For a single-input single-output channel, when no delay exists between the BS and the IoT device, the reference signal at the IoT device is

(1) $$y_{t}=h_{t}x_{t}+z_{t},$$

where *t* is the time slot index, $h_{t}\in C$ is the channel gain, and $x_{t}\in C$ is the reference signal transmitted by the BS. Moreover, $z_{t}\in C$ is the additive white Gaussian noise (AWGN). The IoT device receives a reference signal from the BS and estimates a channel state. Based on the estimated channel information, the IoT device determines whether to report CQI to the BS, where CQI is a 4-bit value that determines the MCS level. If the IoT device does not report its CQI to the BS, the BS uses the last CQI reported by the IoT device. Hence, if there is no feedback delay, the CQI index used by the BS at time slot *t* is updated as follows:

(2) $${\tilde{\mu }}_{t}=\left\{\begin{array}{cc} \mu_{t}, & \mathrm{if the IoT device feeds back}\\ {\tilde{\mu }}_{t-1}, & \mathrm{otherwise}, \end{array}\right.$$

where $\mu_{t}$ is the CQI index reported by the IoT device at time *t*.

In a practical environment, delays and errors exist in the feedback channel, which result in performance degradation. However, for simplicity, we assume no feedback error, but we consider the feedback delay. The delayed channel gain is generated as follows:

(3) $$h_{t,k_{fd}}=\lambda h_{t,k_{fd}-1}+e,$$

where $k_{fd}$ is the feedback delay in a number of time slots, and $h_{t,k_{fd}}$ is the delayed channel gain at time *t*. Moreover, $\lambda =J_{0}\left(2\pi f_{d}T\right)$ is a temporary correlation coefficient, where $J_{0}\left(\right)$ is a zeroth-order Bessel function of the first kind, and $T=\frac{1}{f_{s}}$ is the channel block length. The symbol frequency and the Doppler frequency are represented by $f_{s}$ and $f_{d}$, respectively. *e*, which is independent of $h_{t,k_{fd}-1}$, is distributed with $\mathrm{CN}(0,\rho^{2})$, where the variance of *e* is constrained by $\rho^{2}=1-\lambda^{2}$.

### 2.2. Adaptive Modulation and Coding Scheme

The capacity of an orthogonal frequency-division multiplexing (OFDM) system depends on the modulation method, coding rate, and bit error rate. The modulation method determines the number of bits to transmit per symbol. The coding rate indicates how many actual data bits are included in the transmitted bits. Therefore, the BS dynamically selects the best MCS level according to the channel condition to maximize the capacity. The modulation and coding rate pairs of the 3GPP specification are listed in Table 1 [3].

**Figure 1:**
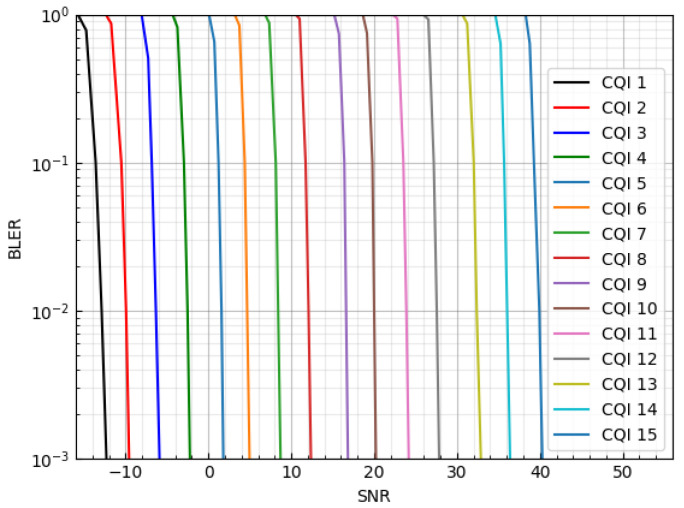
SNR–BLER curve according to MCS levels.

Generally, the BS determines the modulation and coding scheme for downlink as follows: First, an IoT device monitors the channel state based on the channel gain $h_{t}$ at time *t*, and finds the highest CQI index that satisfies the block error rate (BLER) requirement. The SNR–BLER curves are shown in Figure 1 under a Gaussian channel environment in a 5G physical downlink shared channel (PDSCH) link [34]. Second, the IoT device feeds back the selected CQI index to the BS. Finally, the BS determines the MCS level based on the reported CQI index.

## 3. Proposed Lightweight LSTM-Based CQI Feedback

### 3.1. Proposed Lightweight LSTM Model

Considering the limited capacity of IoT devices, we first develop a lightweight LSTM model to reduce the complexity. The reasons for using the LSTM model are as follows: The recurrent neural network (RNN) is widely used for time-series prediction. Because the channel gain is also time-series data, using an RNN is appropriate. However, wireless channels become less correlated over time; therefore, an RNN with long dependencies on past data may not be appropriate. Hence, we used LSTM, which complements the shortcomings of RNNs.

**Figure 2:**
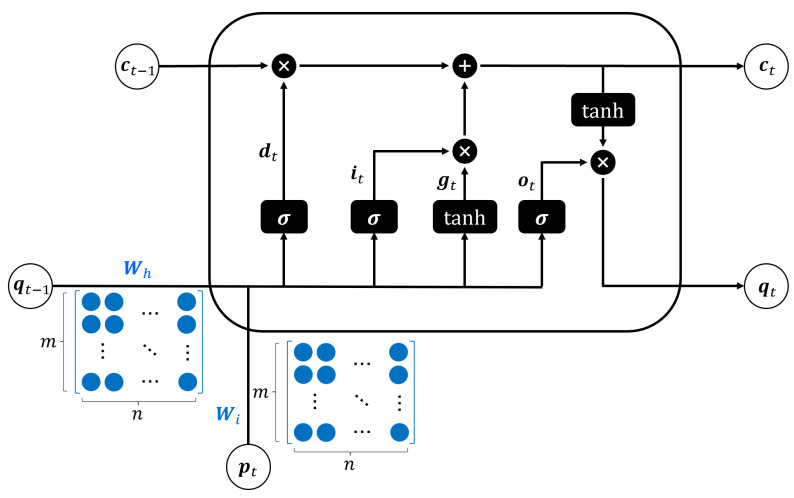
Conventional LSTM cell structure.

Figure 2 shows the conventional LSTM cell structure. All LSTM units consist of a forget gate, an input gate, and an output gate. The mathematical representation of the conventional LSTM structure is given by

(4) $$\begin{array}{ccc} i_{t} & = & \mathrm{sigmoid}(W_{ii}p_{t}+W_{hi}q_{t-1}+b_{i}), \end{array}$$

(5) $$\begin{array}{ccc} d_{t} & = & \mathrm{sigmoid}(W_{if}p_{t}+W_{hf}q_{t-1}+b_{f}), \end{array}$$

(6) $$\begin{array}{ccc} g_{t} & = & \tanh(W_{ig}p_{t}+W_{hg}q_{t-1}+b_{g}), \end{array}$$

(7) $$\begin{array}{ccc} o_{t} & = & \mathrm{sigmoid}(W_{io}p_{t}+W_{ho}q_{t-1}+b_{o}), \end{array}$$

(8) $$\begin{array}{ccc} c_{t} & = & d_{t}*c_{t-1}+i_{t}*g_{t}, \end{array}$$

(9) $$\begin{array}{ccc} q_{t} & = & o_{t}*\tanh\left(c_{t}\right), \end{array}$$

where $p_{t}$ and $q_{t}$ denote the input and hidden states in time step *t*, respectively; W and b denote weight matrices and bias vectors, respectively; sigmoid is an element-wise logistic sigmoid activation function; and ∗ represents element-wise multiplication. Moreover, $i_{t},d_{t}$, and $o_{t}$ denote the input, forget, and output gate, respectively; $c_{t}$ and $g_{t}$ denote the cell state and hidden vector, respectively. Input, forget, and output gates, and the hidden vector can be expressed as the element-wise multiplication and sum of matrices as follows:

(10) $$\begin{array}{ccc} \left[\begin{array}{c} i_{t}\\ d_{t}\\ g_{t}\\ o_{t} \end{array}\right] & = & \left[\begin{array}{c} \mathrm{sigmoid}\\ \mathrm{sigmoid}\\ \tanh\\ \mathrm{sigmoid} \end{array}\right]*\left(W_{i}p_{t}+W_{h}q_{t-1}+b\right), \end{array}$$

(11) $$\begin{array}{ccc} W_{i} & = & {\left[\begin{array}{c} W_{ii},W_{if},W_{ig},W_{io} \end{array}\right]}^{T}, \end{array}$$

(12) $$\begin{array}{ccc} W_{h} & = & {\left[\begin{array}{c} W_{hi},W_{hf},W_{hg},W_{ho} \end{array}\right]}^{T}, \end{array}$$

(13) $$\begin{array}{ccc} B & = & {\left[\begin{array}{c} b_{i},b_{f},b_{g},b_{o} \end{array}\right]}^{T}. \end{array}$$

The conventional LSTM model is either too large or too complex for IoT devices to use. Most of the computational complexity of conventional LSTM models arises from multiplications of matrix–vectors, $W_{i}p_{t}$ and $W_{h}q_{t-1}$. We can reduce the computational complexity of the matrix–vector multiplications by using singular value decomposition (SVD) and applying dimensionality reduction. Suppose a matrix $W\in R^{m\times n}$; the matrix of W is decomposed into $U\in R^{m\times m}$, $\Sigma \in R^{m\times n}$, and $V^{T}\in R^{n\times n}$ by using SVD. One way to reduce the dimensionality of the three matrices, U, Σ, and V, is to set small singular values to zero. If we set *s* small singular values to 0, then we can also eliminate the corresponding *s* columns of U and V [35,36]. Hence, if we choose *r* singular values, which is smaller than *m* and *n*, W is approximated by

(14) $$\begin{array}{ccc} W_{m\times n} & = & U_{m\times m}\Sigma_{m\times n}V_{n\times n}^{T}\\ & \approx & U_{m\times r}\Sigma_{r\times r}V_{r\times n}^{T}\\ & = & U_{m\times r}N_{r\times n}. \end{array}$$

Additionally, the number, *r*, of singular values that we retain determines the energy in Σ [36]. In the proposed lightweight LSTM model, we decompose $W_{h}$ into two matrices, $W_{h1}$ and $W_{h2}$, where the dimension of $W_{h}$ is m×n, the dimension of $W_{h1}$ is m×r, and the dimension of $W_{h2}$ is r×n. Hence, in the conventional LSTM, we should find m×n weights of $W_{h}$ but in the proposed LSTM, we just find (m×r+r×n) weights in $W_{h1}$ and $W_{h2}$. Similar to the decomposition of $W_{h}$, we decompose $W_{i}$ into two matrices, $W_{i1}$ and $W_{i2}$. From (10), the number of matrix–vector multiplications in the conventional LSTM cell is 8·mn times. On the other hand, the number of matrix–vector multiplications in the lightweight LSTM cell is 8·(m+n)r times. Hence, the compression ratio, which is the ratio of multiplications in the conventional LSTM cell and the proposed LSTM cell, is as follows:

(15) $$CR=\frac{(m+n)r}{mn}.$$

**Figure 3:**
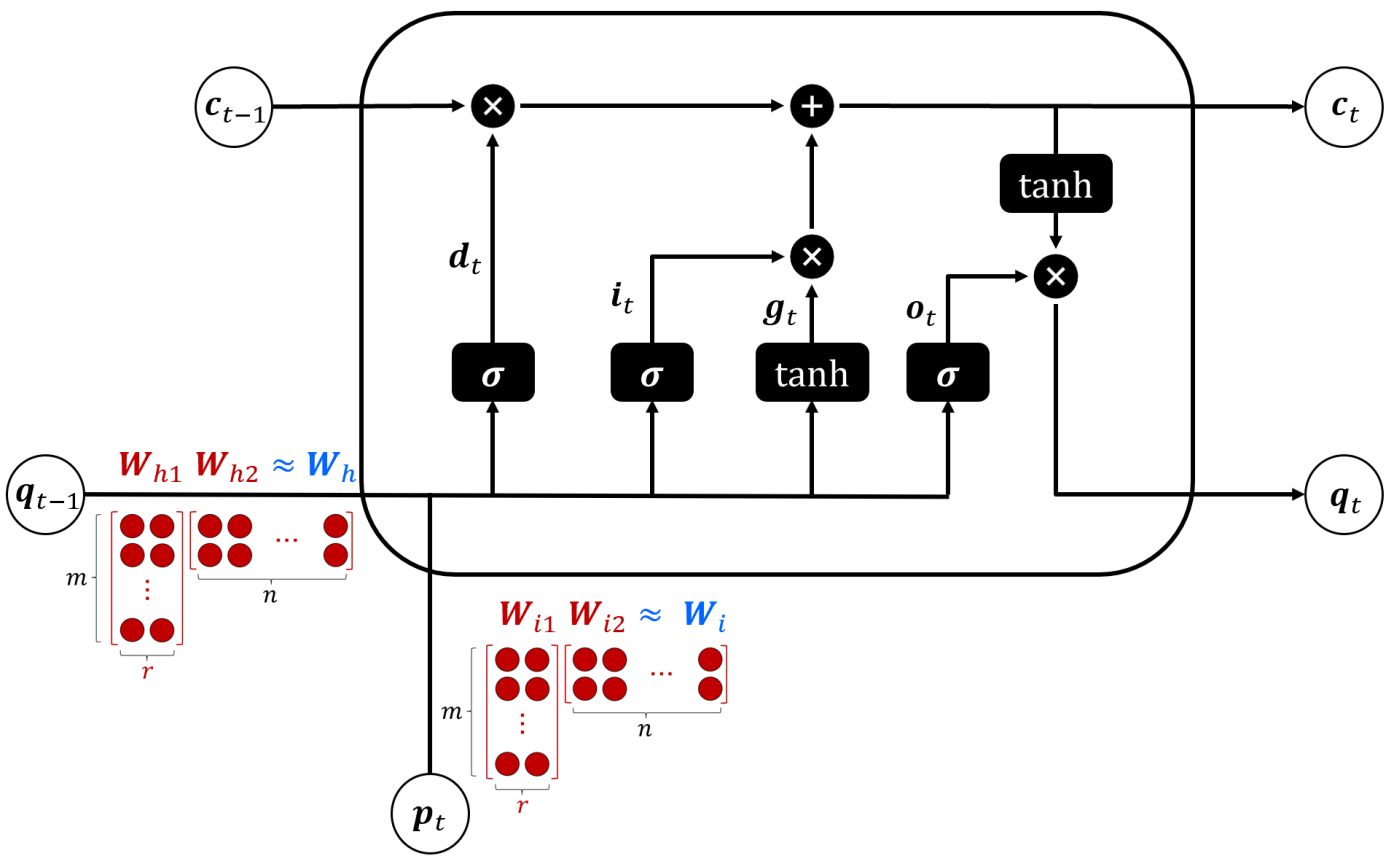
Proposed lightweight LSTM cell structure.

Figure 3 shows the proposed lightweight LSTM structure by applying dimensionality reduction to matrices $W_{h}$ and $W_{i}$ of the conventional LSTM model. Consider the dimension of $W_{h}$ is 5×20. Then, we need to find 5×20=100 weights of $W_{h}$. However, if we reduce the dimensionality by setting r=2, the weight matrix is approximated as $W_{h}\approx W_{h1}W_{h2}$ and we only need to find 50 weights because the dimension of $W_{h1}$ is 5×2 and the dimension of $W_{h2}$ is 2×20. That is, we can reduce the complexity of the LSTM model by half.

### 3.2. Proposed LSTM-Based Aperiodic CQI Feedback

We develop an adaptive CQI feedback scheme based on the proposed lightweight LSTM. The IoT device uses the lightweight LSTM to predict future channel conditions. The proposed lightweight LSTM predicts the channel state in future *M* time slots using the channel state in current and past L−1 time slots at time slot *t*. The function of the lightweight LSTM can be expressed as

(16) $$f\left(\left[h_{t-L+1},h_{t-L+2},...,h_{t}\right],W_{i},W_{h},B\right)=\left[{\hat{h}}_{t+1},{\hat{h}}_{t+2},...,{\hat{h}}_{t+M}\right],$$

where *h* and $\hat{h}$ are the actual and predicted channel state, respectively. The lightweight LSTM should be trained to minimize the error between the predicted channel gains and the actual channel gains. Therefore, we use the mean square error (MSE) loss function as follows:

(17) $$\mathrm{MSE loss}=\frac{1}{M}\sum_{i=1}^{M}{\left|h_{t+i}-{\hat{h}}_{t+i}\right|}^{2}.$$

An IoT device predicts the signal-to-noise ratio (SNR) at future time slot $t+k_{fd}$ as follows:

(18) $${\hat{\gamma }}_{t+k_{fd}}=\frac{PG^{\mathrm{tx}}{\hat{h}}_{t+k_{fd}}G^{\mathrm{rx}}PL}{\sigma^{2}},$$

where $k_{fd}$ is the feedback delay, ${\hat{h}}_{t+k_{fd}}$ is the predicted channel gain, *P* is the transmit power, PL is path loss, $\sigma^{2}$ is noise power, $G^{\mathrm{tx}}$ is the antenna gain at the transmitter, and $G^{\mathrm{rx}}$ is the antenna gain at the receiver. The IoT device aperiodically reports CQI relying on the LSTM-based channel prediction. Because there is the feedback delay of $k_{fd}$, the IoT device needs to report the predicted CQI index, ${\hat{\mu }}_{t+k_{fd}}$, which the BS will use in future time slot $t+k_{fd}$, based on the predicted SNR, ${\hat{\gamma }}_{t+k_{fd}}$. Let ${\tilde{\mu }}_{t+k_{fd}-1}$ denote the CQI index used by the BS at time slot $t+k_{fd}-1$. That is, ${\tilde{\mu }}_{t+k_{fd}-1}$ is the last CQI reported by the IoT device. At time slot *t*, the IoT device feeds back the predicted CQI index if the difference between the CQI index used by the BS, ${\tilde{\mu }}_{t+k_{fd}-1}$, and the CQI index predicted by the IoT device, ${\hat{\mu }}_{t+k_{fd}}$, is above the threshold, ϵ. Hence, with the feedback delay of $k_{fd}$, the CQI index used by the BS at time slot $t+k_{fd}$ is updated as follows:

(19) $${\tilde{\mu }}_{t+k_{fd}}=\left\{\begin{array}{cc} {\hat{\mu }}_{t+k_{fd}}, & \mathrm{if}\;|{\hat{\mu }}_{t+k_{fd}}-{\tilde{\mu }}_{t+k_{fd}-1}|>\epsilon \\ {\tilde{\mu }}_{t+k_{fd}-1}, & \mathrm{otherwise}. \end{array}\right.$$

The above procedure for the lightweight LSTM-based CQI feedback scheme is summarized in Algorithm 1.
**Algorithm 1** Lightweight LSTM-based adaptive CQI feedback.1:Input:   Actual channel gains $\left[h_{t-L+1},h_{t-L+2},...,h_{t},h_{t+1},h_{t+2},...,h_{t+M}\right]$   Threshold ϵ.         /* Training a lightweight LSTM network */2:Build a lightweight LSTM network with *N* input nodes and *M* output nodes.3:Initialize lightweight LSTM network parameters $W_{i},W_{h},B$, and set training epochs.4:**while** epoch < MAX_EPOCH **do**5:    Process $f\left(\left[h_{t-L+1},h_{t-L+2},...,h_{t}\right],W_{i},W_{h},B\right)=\left[{\hat{h}}_{t+1},{\hat{h}}_{t+2},...,{\hat{h}}_{t+M}\right]$6:    Update parameters with loss function $\frac{1}{M}\sum_{i=1}^{M}{\left|h_{t+i}-{\hat{h}}_{t+i}\right|}^{2}$7:    Update epoch ← epoch +18:**end while**         /* Predicting the CQI index */9:Calculate the predicted channel gain with the trained neural network   $f\left(\left[h_{t-L+1},h_{t-L+2},...,h_{t}\right],W_{i},W_{h},B\right)=\left[{\hat{h}}_{t+1},{\hat{h}}_{t+2},...,{\hat{h}}_{t+M}\right]$10:Calculate the predicted SNR ${\hat{\gamma }}_{t+k_{fd}}$11:Calculate the predicted CQI index ${\hat{\mu }}_{t+k_{fd}}$12:**while***t* < MAX_STEP **do**13:    **if** $|{\hat{\mu }}_{t+k_{fd}}-{\tilde{\mu }}_{t+k_{fd}-1}|>\epsilon$ **then**14:        Update ${\tilde{\mu }}_{t+k_{fd}}\leftarrow {\hat{\mu }}_{t+k_{fd}}$15:        Reports the CQI index to the BS16:    **else**17:        Update ${\tilde{\mu }}_{t+k_{fd}}\leftarrow {\tilde{\mu }}_{t+k_{fd}-1}$18:    **end if**19:    Update t←t+120:**end while**

## 4. Results and Discussions

### 4.1. Performance of Lightweight LSTM

We consider two baseline LSTM networks, called LSTM1 and LSTM2, under a channel environment with no feedback delay, to compare the performance of the proposed lightweight LSTM. LSTM1 has the same dimensions of the hidden and weight matrices as the proposed lightweight LSTM, but it has approximately twice the number of multiple-accumulate (MAC) operations than the proposed lightweight LSTM. The dimensions of the hidden and weight matrices of LSTM2 are half those of the proposed lightweight LSTM but has a similar number of MAC operations to the proposed lightweight LSTM. In the proposed LSTM, we set the SVD parameter as r=2. $W_{i}$ is decomposed into $W_{i1}$ and $W_{i2}$, and $W_{h}$ is decomposed $W_{h1}$ and $W_{h2}$. When the dimension of $W_{i}$ (or $W_{h}$) is 5×20, the number of weights to be found is reduced to (5×2+2×20).

**Figure 4:**
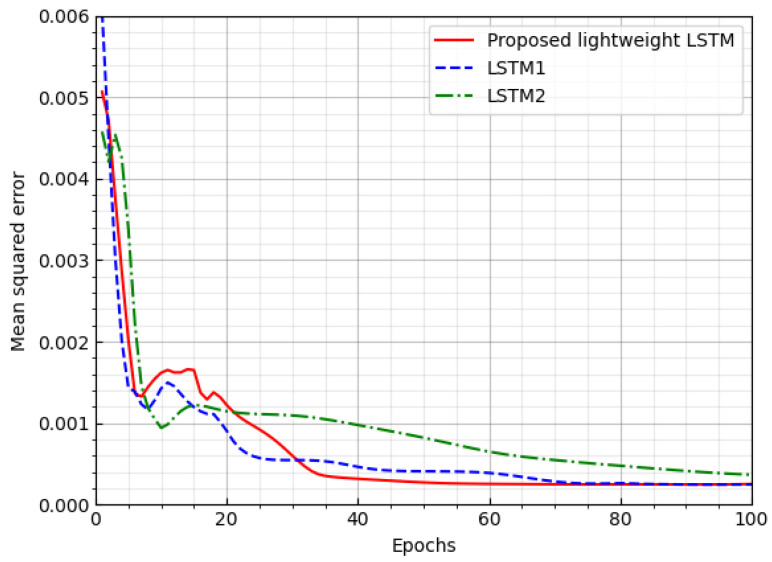
MSE loss of three LSTM networks.

We evaluate the performance of three LSTM networks in terms of the MSE and complexity. Figure 4 shows the MSE loss of the three LSTM networks according to the number of epochs. The proposed LSTM and LSTM1 converges quickly with a lower MSE loss than LSTM2. Table 2 and Table 3 shows the complexity of the three LSTM networks in terms of the number of weights to be found, the number of MAC operations, and the model size. The complexities of the proposed LSTM and LSTM2 networks are similar, being approximately half that of the LSTM1 network. Consequently, the performance of the proposed LSTM model is similar to that of LSTM1, but with half of the complexity.

### 4.2. Performance of Lightweight LSTM-Based CSI Feedback

**Figure 5:**
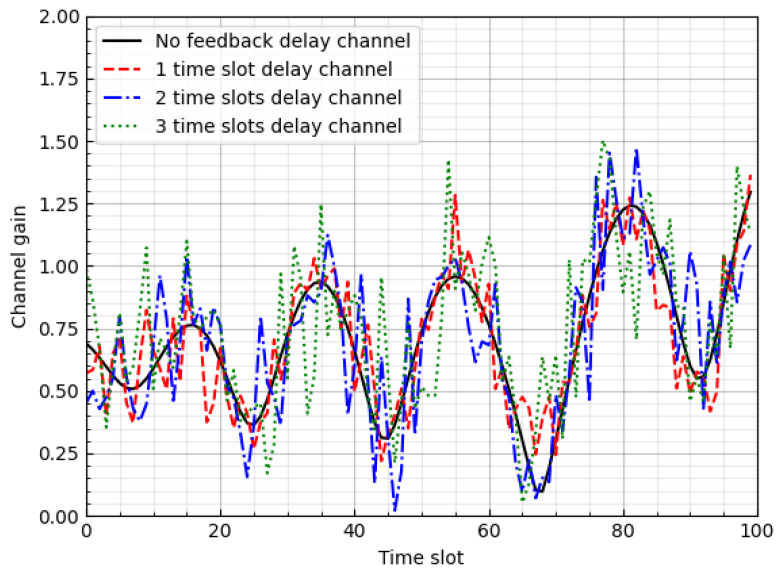
Generated channel gains.

We generated 10,000 Rayleigh fading channel samples using Jakes’ channel model with the parameters listed in Table 4. The delayed channel was then obtained from (3). Figure 5 shows the channel gain over 100 time slots.

Table 5 shows the parameters of the proposed lightweight LSTM. The input and output nodes were set to 5 and 1, respectively. The loss function uses MSE, the number of epochs is 100, the learning rate is 0.01, and the optimizer uses the Adam optimizer.

We evaluate the performance of the LSTM-based CQI feedback scheme in terms of the throughput and the number of feedback transmissions. Here, the throughput at time slot *t* is given by

(20) $$S_{t}=\mathrm{BW}\cdot \log_{2}\left(1+\gamma_{t}\right)\cdot \left(1-\mathrm{BLER}\left({\tilde{\mu }}_{t},\gamma_{t}\right)\right)\;\;[\mathrm{bps}],$$

where BW, which is the bandwidth, is BW=20 MHz, and $\gamma_{t}$ is the current SNR of the channel at time slot *t*. $\mathrm{BLER}({\tilde{\mu }}_{t},\gamma_{t})$ is obtained from Figure 1 when the CQI is ${\tilde{\mu }}_{t}$ and the SNR is $\gamma_{t}$.

**Figure 6:**
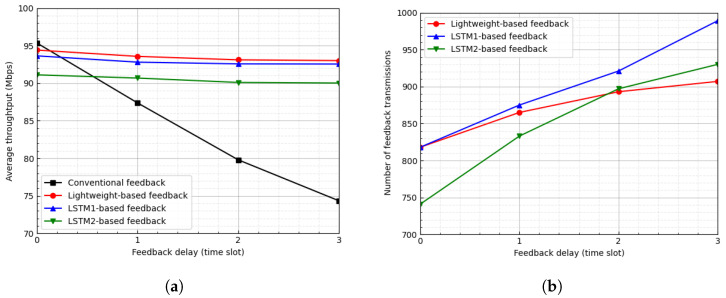
Average throughput and number of feedback transmissions according to feedback delay when ϵ=1. (**a**) Average throughput. (**b**) Number of feedback transmissions.

Figure 6 shows the average throughput and the number of feedback transmissions of CQI feedback schemes according to the feedback delay when the threshold is ϵ=1. Figure 6a shows the average throughput of four kinds of CQI feedback schemes according to the feedback delay. When feedback is not delayed, the conventional CQI feedback scheme produces the highest throughput due to the accuracy of the channel state because the IoT device reports its CQI to the BS for every time slot. However, as the feedback delay increases, the reported CQI information is outdated; therefore, the throughput decreases due to the inaccuracy of the channel state. The three LSTM-based CQI feedback schemes produce relatively constant throughput regardless of the feedback delay because they predict the future channel gains. The LSTM1-based feedback scheme outperforms the LSTM2-based feedback scheme owing to the accuracy of the LSTM model due to its complexity. The proposed lightweight LSTM-based feedback scheme outperforms the other LSTM-based feedback schemes despite having the lowest complexity. Figure 6b shows the number of feedback transmissions over 3000 time slots. In the conventional CQI feedback scheme, the number of feedback transmissions is equal to the number of elapsed time slots because the IoT device periodically reports its CQI to the BS every time slot. In LSTM-based feedback schemes, as the feedback delay increases, the number of feedback transmissions increases relatively slowly; moreover, the number of transmissions in the proposed scheme increases more slowly. In particular, for ϵ=1, when feedback is not delayed, the proposed scheme degrades the average throughput by approximately 1% and reduces the number of feedback transmissions by approximately 72%.

**Figure 7:**
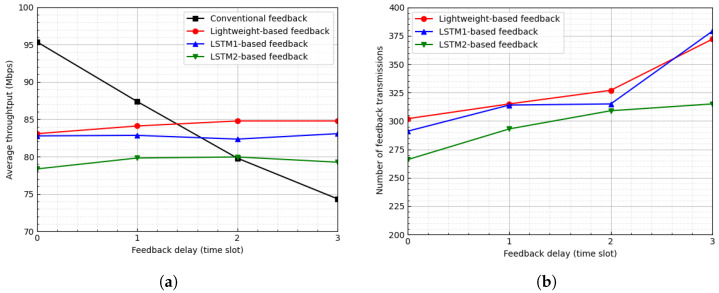
Average throughput and number of feedback transmissions according to feedback delay when ϵ=2. (**a**) Average throughput. (**b**) Number of feedback transmissions.

**Figure 8:**
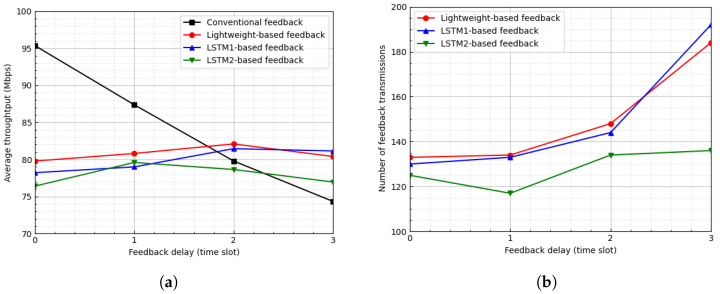
Average throughput and number of feedback transmissions according to feedback delay when ϵ=3. (**a**) Average throughput. (**b**) Number of feedback transmissions.

Figure 7 and Figure 8 show the average throughput and the number of feedback transmissions when the threshold is ϵ=2 and 3, respectively. In the LSTM-based feedback schemes, as the value of ϵ increases, the number of feedback transmissions decreases, which results in an inaccurate channel state. Hence, as the value of ϵ increases, the average throughput of the LSTM-based feedback schemes degrades. In Figure 7, for ϵ=2, when the feedback delay is one time slot, the proposed scheme degrades the average throughput by approximately 3.74% and reduces the number of feedback transmissions by approximately 89.48%. Additionally, in Figure 8, for ϵ=2, when the feedback delay is two time slots, the proposed scheme degrades the average throughput by approximately 2.88% and reduces the number of feedback transmissions by approximately 95.06%.

## 5. Conclusions

In this paper, we proposed a lightweight LSTM-based adaptive CQI feedback scheme for IoT devices, where the IoT device aperiodically reports its CQI on the basis of an LSTM-based channel prediction. We developed a lightweight LSTM by using singular value decomposition (SVD) and applying dimensionality reduction. The complexity of the developed LSTM is approximately two times lower than that of the conventional LSTM without sacrificing performance. In the proposed LSTM-based CQI feedback scheme, the IoT device predicts the future channel states relying on the developed lightweight LSTM and determines whether it reports CQI to the BS according to the difference between the last reported CQI and the predicted CQI. In comparison with the conventional periodic CQI feedback scheme, the performance of the proposed lightweight LSTM-based CQI feedback scheme only degrades approximately 4%, but the number of feedback transmissions is reduced by more than approximately 72%.

In practice, IoT devices for sensing and tracking are typically low-cost systems, requiring reduced hardware and software complexity. In comparison with the convention LSTM model, the proposed lightweight LSTM model enables the use of lightweight IoT devices by reducing the complexity by half without deteriorating performance. Moreover, the proposed LSTM-based aperiodic CQI feedback significantly decreases the number of feedback transmissions, resulting in reduced uplink interference. However, because the proposed CQI feedback scheme uses LSTM to predict the future channel state, each IoT device requires learning time and memory to store weights.

For simplicity, no feedback error was assumed in this paper, but for further study, this work can be extended to channel environments with feedback error. Moreover, it is necessary to find an appropriate value of *r* that determines the SVD dimensionality reduction. Because the number of singular values that we retain in the SVD dimensionality reduction determines the energy, we need to find an appropriate value that compromises on the balance between the channel prediction accuracy and the complexity reduction for different channel environments. 

## Figures and Tables

**Table 1:** Modulation and coding schemes.

CQI index, μ	Modulation	Code Rate (×1024)	Efficiency
0		out of range	
1	QPSK	78	0.1523
2	QPSK	120	0.2344
3	QPSK	193	0.3770
4	QPSK	308	0.6016
5	QPSK	449	0.8770
6	QPSK	608	1.1758
7	16QAM	378	1.4766
8	16QAM	490	1.9141
9	16QAM	616	2.4063
10	64QAM	466	2.7305
11	64QAM	567	3.3233
12	64QAM	666	3.9023
13	64QAM	772	4.5234
14	64QAM	873	5.1152
15	64QAM	948	5.5574

**Table 2:** Number of weights in LSTM networks.

LSTM Model	Hidden Dimension	Number of Weights to Be Found in $W_{i}$ (or $W_{h}$)
LSTM1	20	5×20
LSTM2	10	5×10
Proposed lightweight LSTM	20	(5×2+2×20)

**Table 3:** Complexity of LSTM networks.

LSTM Model	Number of MAC Operations	Model Size
LSTM1	2303 k	13.40 kB
LSTM2	1183 k	7.35 kB
Proposed lightweight LSTM	1160 k	7.34 kB

**Table 4:** Channel generation parameters.

Parameter	Value
Channel model	Rayleigh fading
Number of channel samples	10,000
Symbol frequency	5 kHz
Doppler frequency	150 Hz
Transmitter power	24 dBm
Noise figure	2 dB
Thermal noise	−118.4 dB
Path loss	120 dB

**Table 5:** Parameters of lightweight LSTM.

Parameter	Value
Input nodes, *L*	5
Output nodes, *M*	1
Hidden dimension	20
Cost function	Mean square error
Learning rate	0.01
Optimizer	Adam
Epoch	100
Number of training dataset	7000
Number of testing dataset	3000

## Data Availability

Not applicable.
